# Uncovering the Early-Stage Intercalation Mechanism in Graphite-Based Anode Materials

**DOI:** 10.1021/acsami.5c04287

**Published:** 2025-05-28

**Authors:** Jafar Azizi, Axel Groß, Holger Euchner

**Affiliations:** † Institute of Theoretical Chemistry, Ulm University, Ulm D-89081, Germany; ‡ Institute of Physical and Theoretical Chemistry, University of Tübingen, Tübingen 72076, Germany

**Keywords:** anode material, intercalation mechanism, graphite, alkali metal, DFT

## Abstract

Graphite and graphite derivatives, the standard anode materials for Li-ion batteries, are also of great interest for post-Li-ion technologies, such as potassium-ion batteries. However, certain aspects of the intercalation process in these systems, as well as the resulting consequences, still require a deeper understanding. In particular, the first steps of K intercalation in graphitic systems, i.e., at low concentrations, are fundamentally different from the case of Li. Herein, we use density functional theory to elucidate the early-stage intercalation of K in graphitic materials by seeking comparison to the behavior of Li and Na. Our results show the crucial role of the competition between the interlayer van der Waals interaction and the alkali metal−carbon bond formation for the initial stages of intercalation of large alkali metal atoms. As a consequence, and in contrast to the case of Li, K intercalation becomes energetically unfavorable at low concentrations. This is a significant finding, which can explain the origin of the differences observed for Li and K intercalation in graphitic materials. Hence, we identify the first steps of K intercalation as potential reasons for performance loss and battery failure and show that heteroatom doping can open pathways for solving these issues.

## Graphical abstract

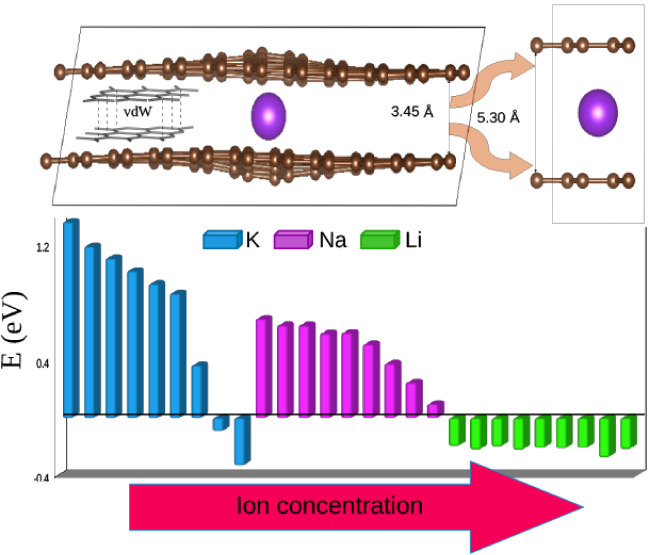

## 1 Introduction

The exceptional mechanical, electrical, and thermodynamic properties of two-dimensional (2D) van der Waals (vdW) materials, which are typically not observed in their three-dimensional (3D) counterparts, have sparked a lot of attention in many fields of research. In particular, 2D materials such as graphite and its derivatives, as well as dichalcogenides, have emerged as promising electrode components in batteries and energy storage systems.^1–5^

Graphite and derivatives thereof are still the standard option for anodes in commercial Li-ion batteries (LIBs).^2,6,7^ It is well known that graphite can intercalate alkali metal (AM) atoms between its layered structure, with half-filled carbon p_z_ orbitals that are perpendicular to the graphitic planes and can interact with the AM s-orbitals.^8,9^ Due to its small size and the associated exceptional behavior, Li intercalates easily into graphitic compounds. For larger AM atoms, on the other hand, the competition between vdW interaction and AM-C bond formation can be challenging for small AM concentrations.^10–13^

The working principle of LIBs on the anode side is based on the intercalation and deintercalation of Li atoms between the layers of graphite. Starting from the pioneering work of Hérold in the 1950s^14^ lithium graphite intercalation compounds (Li-GICs) have been extensively investigated.^15–18^ Their structural evolution during intercalation/deintercalation was investigated using different experimental techniques such as X-ray diffraction (XRD), Raman scattering or nuclear magnetic resonance.^19–24^ Li-GICs exhibit different compositions and crystal structures^25–28^ that can be described by the so-called staging mechanism,^29–32^ which refers to the periodic stacking of Li layers between the graphitic planes. The resulting structures are denoted as stage-n compounds, where the index n denotes the number of graphene layers stacked between the (filled) intercalant layers.^25,33,34^ According to earlier studies^25,35^ GICs undergo a shift from AB- to AA-stacking with increasing AM concentration, when sufficient binding energy is provided to overcome the AB-AA transition. These findings were recently also confirmed for defect-containing GICs.^36^ For Li, thermodynamically stable GICs with increased AM content exist up to a maximum Li content equivalent to an LiC_6_ stoichiometry, corresponding to stage-I GICs.^2^ Here, the intercalation process, including the observation of different stage-n compounds, is well-understood and has been investigated by a variety of experimental techniques such as X-ray diffraction or Raman scattering.^23^ Na-GICs, on the other hand, have only been observed with a low Na concentration. While the amount of electrochemically intercalated Na depends for instance on the electrolyte composition or defect concentration, intercalation limits of NaC_64_ or less have been reported.^39–44^ The underlying thermodynamic instability of the intercalation process for reasonable amounts of Na therefore renders graphite unsuited as anode material for sodium ion batteries (NIBs).^25,36,45–47^ Several studies have addressed this issue, explaining why—despite the chemical similarities of Li and Na—graphite does not properly work for sodium intercalation.^12,33^ In fact, computational investigations have shown that the competition of AM-graphite coupling strength and AM ionization energy determine the intercalation process.^48^ Interestingly, due to its small size and additional covalent contributions to the Li–C bonds it is, however, rather Li that shows unexpected behavior.^25^ For the case of K, stable GICs with significant AM content, such as KC_8_ are observed. The electrochemical intercalation of potassium into graphite has also been extensively studied by different groups and methods, demonstrating a phase evolution and staging behavior that is comparable to, but distinct from, the case of Li.^35,49–52^ For instance, the stage-I compound KC_8_, as well as stage-II and stage-III compounds with KC_16_ and KC_24_ stoichiometry have been experimentally observed.^52–54^ Moreover, recent studies also have indicated additional (meta-) stable configurations for intermediate K concentrations.^35^ While larger differences in the intercalation mechanism have been observed for low AM concentrations^35^ most studies focus on GICs with increased AM concentration. Hence, the early steps of intercalation for K in graphite and the atomistic origin as well as the consequences of the differences compared to Li have not been addressed so far. In the present work, we systematically investigated the structure and energetics of the first steps of the AM intercalation process in GICs, by means of density functional theory (DFT) calculations. Based on the computed formation energies, we evaluated the stability of the AM intercalation for decreasing AM concentrations. For this purpose, graphite bulk structures based on different numbers of stacked layers and lateral system sizes were constructed. In order to gain a better understanding of the origin and consequences of the observed differences in early-stage K and Li intercalation, we carefully compared the results for Li and K. Furthermore, to better identify potential trends, Na intercalation was also considered.

Regardless of the stability of the intercalation compounds at high AM concentrations, our simulations show that the initial insertion step is dominated by a competition between the vdW interaction and the intercalation energy. At low concentrations, this competition can—depending on the intercalant—result in peculiarities in the intercalation process. In fact, for low concentrations of large intercalants such as potassium, the graphitic layers around the AM atoms become strongly curved, resulting in a different initial intercalation mechanism compared to that observed, for instance, with the small Li atom.

Furthermore, the AM diffusion kinetics were investigated for low AM contents, focusing on the impact of changes in local geometry. Our results show that an increasing lateral system size, corresponding to a lower intralayer AM concentration, affects the diffusion of AM atoms , especially for large atoms like K—an effect that has so far not been addressed in the literature.

## 2 Methods

To investigate the initial stage of AM intercalation, graphite-based model systems have been studied using periodic density functional theory (DFT). For modeling graphitic domains, the investigated bulk systems have been constructed as stacks of graphitic layers. First, different lateral supercell sizes (AMC_*N*×_ *_N_*_×2_ and AMC_*N*×__*N*__×3_, AM = Li, Na, and K, and *N* = 2...10) have been constructed to investigate the impact of decreasing AM concentrations. In addition, decreasing AM contents have been probed via structures with increasing layer thickness, corresponding to 2 × 2 × *N* supercells (with *N* = 2...8).

All simulations were performed using the Vienna Ab Initio Simulation Package (VASP),^55^ employing the Projector Augmented Wave (PAW) approach.^56^ Exchange and correlation were described using the optPBE functional, which includes a nonlocal correction scheme to account for van der Waals interactions.^57^ The convergence criterion for the self-consistent field (SCF) cycle was set to 1 × 10^–7^ eV, while the geometry was optimized until the remaining forces were less than 1 × 10^–3^ eV/Å. Each structure was optimized with respect to the lattice constant and atomic positions, applying a plane wave cutoff of 600 eV. The structures based on the 2 × 2 × 2 (corresponding to C_16_) supercell were computed with a 10 × 10 × 6 k-point mesh, while the other system sizes under investigation were optimized with a corresponding k-point resolution. To assess AM migration, the Nudged Elastic Band (NEB) method^58,59^ was applied, typically using five images along the reaction path. For all considered models, rather large system sizes were chosen to ensure negligible interaction between the periodic images of the migrating AM atoms.

## 3 Results and Discussion

### 3.1 Energetics of the Intercalation Process

In order to investigate the thermodynamic stability of early-stage AM intercalation, two- and three-layer-based bulk systems with different AM atom concentrations have been considered, as depicted in Figures 1 and S1. For these systems, the energetic stability of an intercalated single AM atom was investigated by calculating the intercalation energy with respect to the AM-free system:

1 $$E_{\mathrm{int}}=E_{G+\mathrm{AM}}-\left(E_{G}+E_{\mathrm{AM}}\right)$$

**Figure 1:**
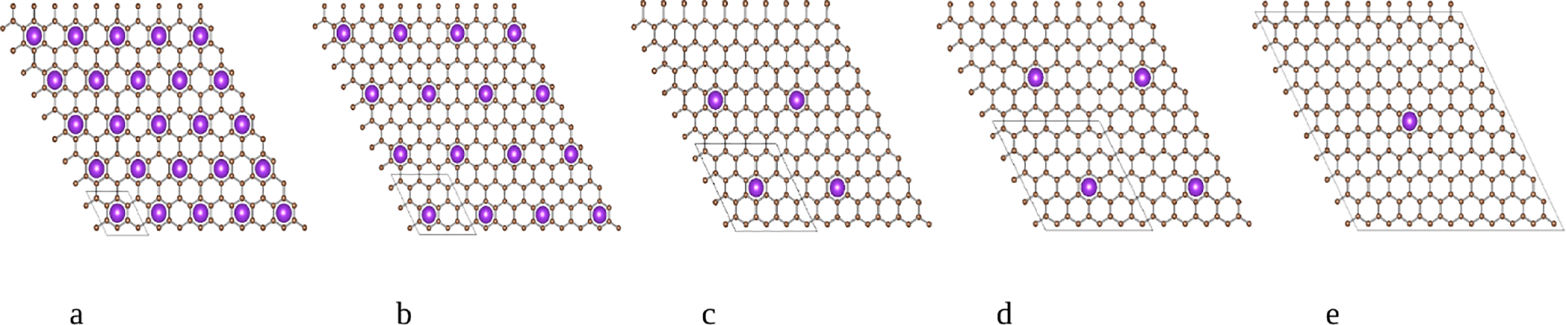
Schematic representation (projected along the *c*-axis) of AM intercalation compounds with (a) KC_16_, (b) KC_36_, (c) KC_64_, (d) KC_100_, and (e) KC_400_ stoichiometry. The simulation unit cells are indicated in black.

Here, *E*_G+AM_ is the total energy of the graphitic system after the insertion of one AM atom. *E*_G_ is the energy of the AM-free layers, and *E*_AM_ is the energy of the AM in the bulk metal phase. In the case of Li, intercalation at low concentration is energetically favorable, yielding an energy gain of ∼−0.2 eV for different AM concentrations in a two-layer bulk system, as depicted in Figure 3a and Table 1. Slightly varying intercalation energies have been obtained for three-layer-based bulk structures (see Table S1). Note that the stronger the intercalation in a particular anode material—i.e., the more negative the intercalation energy—the smaller the open-circuit voltage of the corresponding battery. However, a certain driving force is needed to avoid metal plating at the anode. The fact that Li intercalation is energetically favorable is, among other factors, a consequence of the small size of the Li atom, resulting only in local distortions around the intercalated ion, with a range that is already covered by small supercell sizes. Here, the small observed fluctuations in the intercalation energy for increasing system size are due to the matching conditions of the introduced distortion with the periodicity of the cell. Regarding K intercalation, on the other hand, significant differences are observed in comparison to Li. The K atom intercalation also becomes favorable for higher concentrations; however, for increased lateral system size and hence low AM contents—in contrast to the case of Li—it becomes largely unfavorable, as depicted in Figure 3a and Table 1. The corresponding results for the three-layer-based bulk system are given in Table S1. Finally, for Na, it is known that no significant amounts of ions can be intercalated, with the intercalation of rather small fractions already becoming thermodynamically unstable. As in the case of K, at very low concentrations, the further decrease of the Na content turns the intercalation more and more unfavorable (see Figure 3a and Table 1). So, while Li intercalation is essentially independent of the concentration—in the low concentration limit—decreasing the K content to roughly KC_64_ (or less) results in a tremendous change in the intercalation energy for the two-layer-based bulk system. For Na, the same effect, albeit with a somewhat less pronounced change, is observed. Hence, even at very low concentrations, there is a significant impact of ion type (and size) on the intercalation process.

**Table 1:** Intercalation Energy E_*int*_ (in eV) for Li, Na, and K Atoms in the Two-Layer-Based Bulk Model System with Respect to Different Supercell Sizes/Atom Concentrations

Models	C_16_	C_36_	C_64_	C_100_	C_144_	C_196_	C_256_	C_324_	C_400_
Li	–0.21	–0.27	–0.21	–0.21	–0.21	–0.22	–0.20	–0.22	–0.19
Na	0.10	0.25	0.38	0.61	0.59	0.59	0.64	0.64	0.76
K	–0.33	–0.10	0.37	0.87	1.22	1.02	1.11	1.19	1.47

Indeed, the intercalation of small fractions of Na and K atoms significantly affects the geometry of the system and results in a local curvature around the intercalants, as shown in Figure 2. Taking a closer look at the geometry of a KC_400_ model structure—i.e., at very low K concentrations—reveals that far from the intercalant site, the layer distance reaches a value of 3.44 Å, which almost corresponds to that of pristine graphite. However, in the vicinity of the intercalant, the system is strongly curved (see inset of Figure 3). In general, decreasing the AM concentration results in a strong, localized curvature of the graphitic layers around the intercalated atoms, which is caused by the competition between AM intercalation and interplane vdW interactions. While the vdW forces attempt to keep the neighboring graphitic layers at their equilibrium distance, the intercalants aim to increase the spacing between the layers. The consequence of these competing forces is that the system minimizes its energy by introducing a curvature into the graphitic layers in the vicinity of the intercalated ion. The strength of this curvature depends on the ionic radii of the intercalant, thereby showing a more pronounced effect for larger AM atoms.

**Figure 2:**
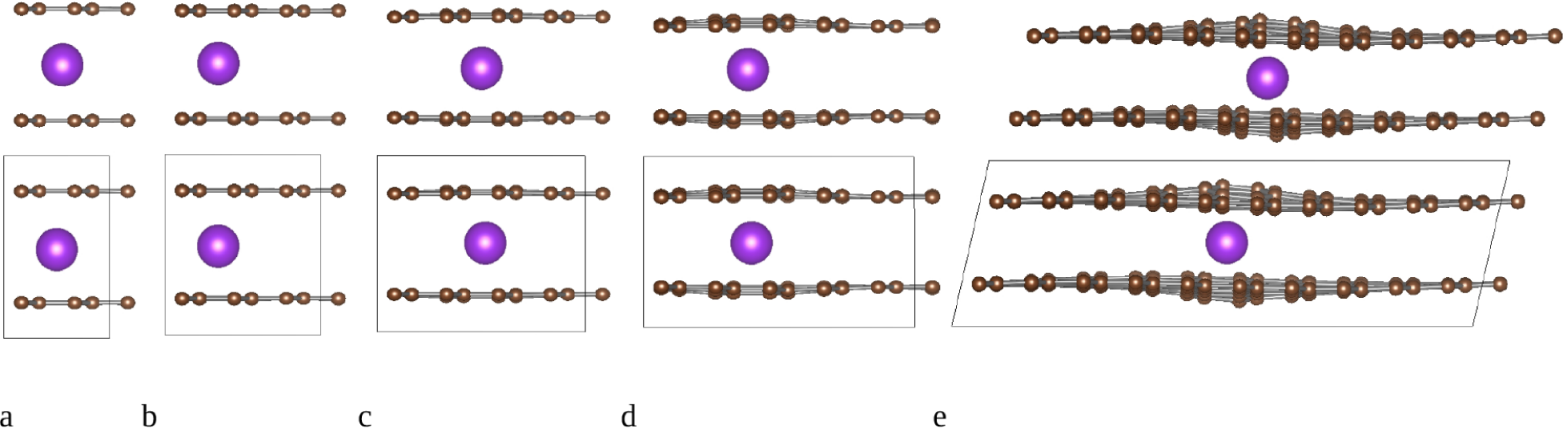
Schematic representation (side view) of the different supercell sizes used to model the AM atom intercalation, showing the distortions introduced by K atoms for (a) KC_16_, (b) KC_36_, (c) KC_64_, (d) KC_100_, and (e) KC_400_. The resulting changes in interlayer distance are given in Figure 4b,c; corresponding results for the three-layer based bulk supercells are shown in Table S1 and Figures S1 and S2.

**Figure 3:**
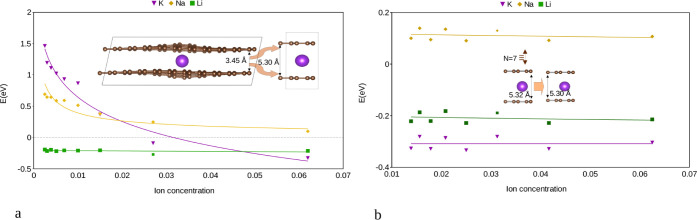
Intercalation energy *E*_int_ (in eV) for single AM (Li, Na, and K) atoms as obtained for the two-layer based bulk system. (a) Intercalation energy with respect to the different lateral supercell sizes and hence different AM concentrations, (b) intercalation energy for systems with a different number of stacked layers based on an AMC_16_ cell. The insets show the change in the layer distance for the case of K intercalation. The solid lines serve as guides for the eye.

Finally, it is crucial to point out again that while the intercalation of K atoms is unfavorable at low concentrations, K intercalation compounds become energetically stable at a higher AM content. Thus, a certain K concentration per unit cell—in the two-layer-based system somewhere between KC_64_ and KC_36_)—stabilizes the intercalation, meaning that the energy gain due to K–C bonds compensates for the energy penalty caused by a decreased vdW interaction (see Figure 3a and Table 1). This indicates that a certain K concentration between two graphitic planes is necessary to stabilize the intercalation process.

To further validate this assumption, we have investigated the impact of gradually decreasing the AM concentration by increasing the number of carbon layers in the underlying bulk supercell, again starting from the two-layer-based bulk structure. To investigate the AM intercalation, the starting stoichiometry of AMC_16_, corresponding to one completely filled AM layer and one empty AM layer (stage II compound), was chosen. By adding further (empty) carbon layers to the bulk supercell—i.e., the AM concentration in the filled layers remains high—the intercalation energy is, in all cases, found to reach a constant value (see Figure 3b). It should be noted that the AMC_16_ compounds—as is well-known from the literature—adopt an AA stacking sequence. On the other hand, empty graphitic layers that are added to increase the system size were chosen to follow an AB stacking sequence. Due to the periodic boundary conditions, this means that simulations with an odd number of graphitic layers will have no empty AA-stacked layers, while those with even layer numbers are forced to have one—energetically less favorable—empty AA-stacked layer. Hence, periodic-like fluctuations of the formation energy are observed, while the latter does not show considerable changes with an increasing number of carbon layers (see Figure 3b). This indeed means that the AM concentration per layer is the crucial factor that determines the thermodynamic stability of the K intercalation. In other words, low K concentrations can only be stably intercalated by locally high K contents within one layer. This differs from the case of Li and leaves the question, how the K intercalation process can be initiated, to some extent open. The energetically unfavorable intercalation of Na and, in particular, K atoms in graphitic systems at low AM concentrations means that, instead of entering the anodes, the AM atoms would rather form metallic deposits on the anode surface. This indicates that plating would occur, which could then lead to dendrite growth and fire hazards in a battery.^60^ Indeed, plating on graphitic anodes has been observed to represent a considerable safety issue in sodium-ion batteries^61^ but especially in potassium-ion batteries.^62^ Together with the experimentally observed increased resistance^35^ this can hence be understood as a consequence of the unfavorable energetics at low K concentrations. Consequently, an in-depth understanding of the early stages of AM intercalation is crucial for improving carbon-based anodes for potassium-ion batteries (KIBs). In the following, we therefore further investigate the reasons for the differences in the (early stage) AM intercalation mechanism as well as the corresponding diffusion kinetics. Finally, we propose strategies to mitigate the energetically (partially) unfavorable Na and K intercalation in graphitic anodes, which may help to promote NIBs and KIBs as efficient and more sustainable alternatives to LIBs.^63^

### 3.2 Cavity Formation Energy

To further elucidate the differences in the intercalation process for Li, Na, and K, we have divided the latter into two steps. The first step corresponds to the formation of the cavity created by the respective AM atom. By calculating the penalty for the formation of this cavity, the energy needed to compensate the vdW interaction can be deduced (see Figure 4a). In principle, this energy penalty can be understood as a kind of defect formation energy, which can be computed via the following expression:

2 $$E_{\mathrm{def}}=E_{\mathrm{CG}}-E_{\mathrm{PG}}$$

where *E*_CG_ and *E*_PG_ represent the total energy of the curved and pristine graphite. As already discussed in the previous sections, the created cavity is dependent on the size of the intercalant. Consequently, the defect formation energies show a significant variation based on the alkali metal and its size. Hence, compared to Li, the Na and K cavities result in a much higher defect formation energy. While the defect formation energy for Li is almost constant with respect to the lateral supercell size, the defect formation energy for Na increases for larger supercells (see Figure 4a). Finally, the defect formation energy of the cavity originating from K intercalations is found to be even higher than that of Na, as expected from its larger ionic radius. For both cases, the defect formation energies are expected to reach a constant value at low concentrations (i.e., for even larger system sizes), but this point does not seem to be reached yet. Furthermore, by determining the maximum and minimum layer spacing in the considered model system, the extension of the cavity can be quantified. For the case of Li, the cavity approaches a constant size when the lateral dimensions of the supercell increase (see Figure 4b,c), which is in accordance with the defect formation energy being constant. In principle, the same behavior can be expected for Na and K; however, the extension of the cavity is much larger, and as already seen for the defect formation energy, this point is not fully reached yet.

**Figure 4:**
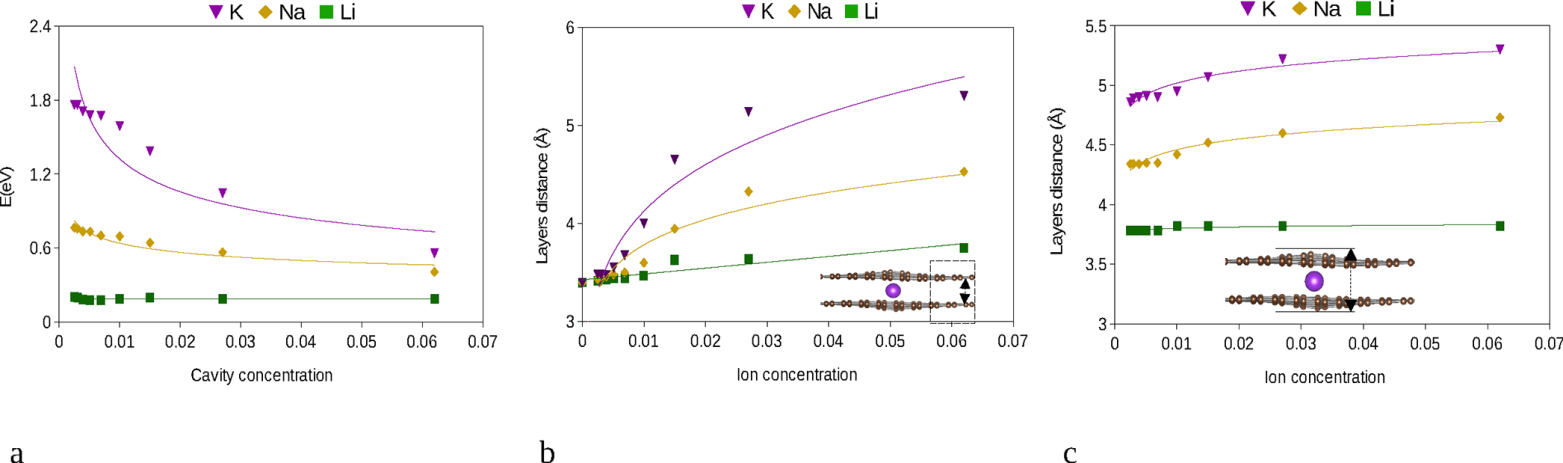
Graphite layer distance and defect formation energy for different super cell sizes of the two-layer based bulk system (AMC_*N*__×_ *_N_*_×2_, AM = Li, Na, and K, *N* = 2, 3, ..., 10). (a) Defect (curvature) formation energy for different super cell sizes, (b) graphite layers distance far from the intercalant and (c) layer distance at the intercalant site.

### 3.3 Diffusion Kinetics

Apart from the stable intercalation of the AM atoms, their kinetics is also of great importance for battery applications. Hence, in order to gain an atomistic understanding of the AM kinetics during the charging/discharging process, the energy barriers for K diffusion at different ion concentrations, again focusing on the early-stage intercalation, were calculated and compared to the case of Li by using the Nudged Elastic Band (NEB) method (see Figure 5). As with the intercalation energies, two types of structures, based on increasing lateral size and the number of stacked layers, were taken into consideration in order to comprehend the diffusion mechanism at low AM concentration. To determine the impact of increasing lateral system size (decreasing AM concentration) and hence increasing AM–AM distance, the three-layer-based bulk system was considered. Here, the choice of a three-layer-based supercell with two empty layers was made to ensure that no spurious effects were introduced by periodic boundary conditions.

**Figure 5:**
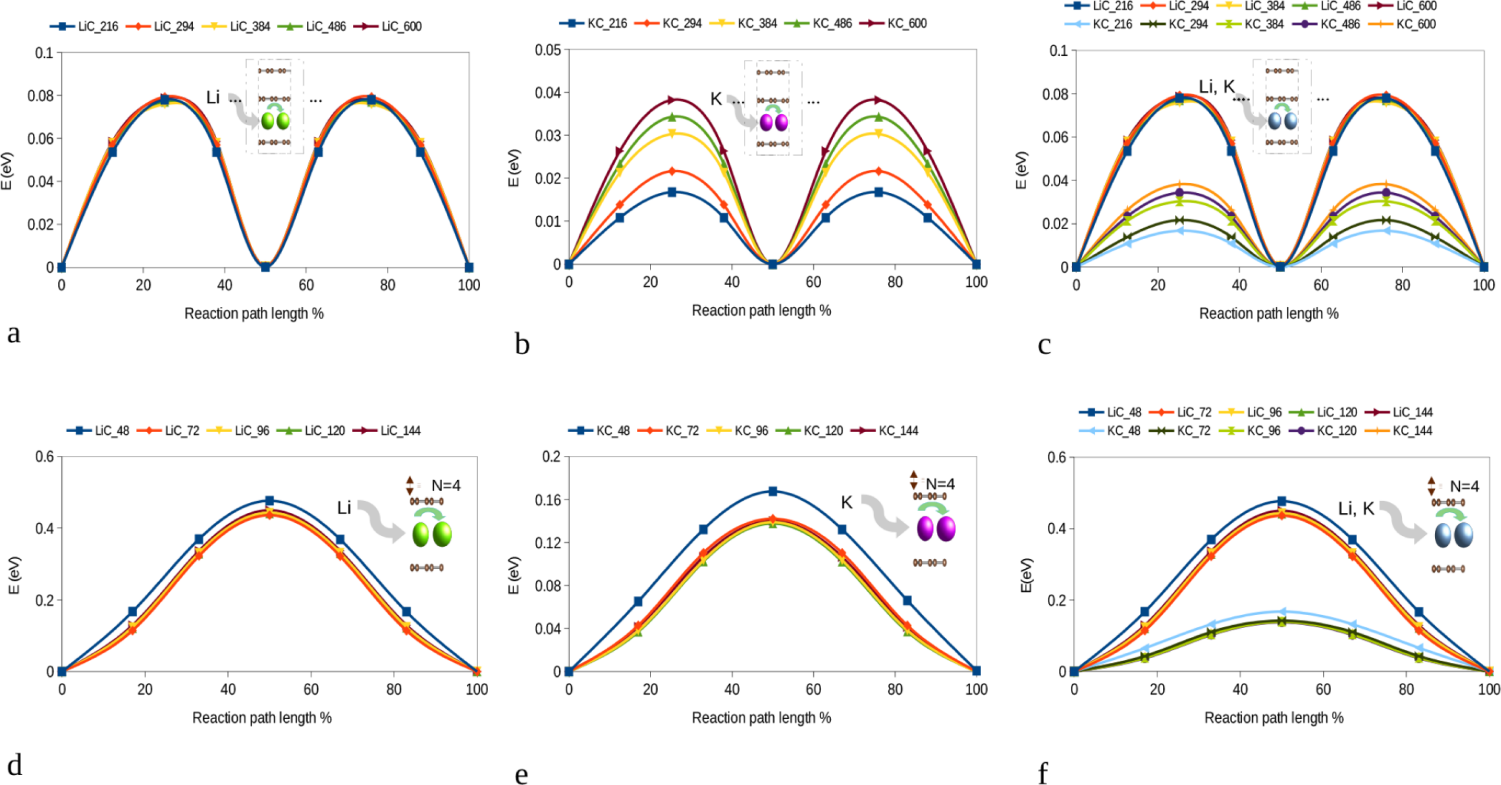
Minimum energy path for (a) Li and (b) K diffusion in a three-layer-based bulk system with different lateral supercell sizes (AMC_*N*__×_ *_N_*_×__3_, AM = Li, K, and *N* = 6–10). Due to the low AM concentration, the AM containing layers are AB stacked. For better comparison, panels (a) and (b) are merged in (c). (d) Li and (e) K diffusion in the AMC_48_ unit cell and for different numbers of graphite layers on top of each other. For better comparison, (d) and (e) are merged in (f). Due to the increased AM concentration per layer, the AM containing layers are AA stacked.

As the AB-stacked graphite is more favorable for low concentrations, we considered a stacking sequence in which the two layers containing the AM atoms exhibit AB-stacking. It should be noted that, while in AA-stacked graphite the AM atoms are located above or below hollow sites of both the upper and lower layers, in AB-stacking the AMs are located at the hollow site of one graphitic layer and at a top site with respect to the other. For the investigated low concentrations, the Li migration barriers amount to less than 0.1 eV and are essentially independent of the lateral system size (see Figure 5a). For K, the migration barriers are even further reduced but show a slight dependence on the lateral system size. The observed impact of system size (and hence K concentration) on the K kinetics can be understood as a consequence of the extension of the introduced distortions. For higher in-plane AM concentrations, the increased lattice spacing around the AM atom results in more space and hence a lowering of the diffusion barriers (see Figure 2). For the sake of comparison, we have also investigated the same scenario for the AM atoms residing between AA-stacked layers. Here, the findings are qualitatively the same, with the Li diffusion barriers being largely independent of the lateral size, whereas for the case of K, the barriers again showed a slight increase with the system size (see Figure S3). Yet, it must be pointed out that the diffusion barriers for the AA-stacked layers are significantly increased, amounting to 0.395–0.41 eV and 0.15–0.22 eV for Li and K, respectively. Thus, the diffusion of Li and K is strongly enhanced as long as AB-stacking is dominant.

Finally, the impact of the number of empty graphitic layers on the diffusion barriers of the intercalants was investigated. In this scenario, low AM concentrations are again obtained by increasing the number of graphitic layers while considering one layer with a particular AM content that has already transformed to AA-stacking. Here, starting from a two-layer-based bulk system with LiC_48_ and KC_48_ stoichiometry, no significant changes are observed for an increasing number of graphite layers as soon as more than three layers are considered (see Figure 5b). The migration barriers for Li and K amount to 0.43 and 0.14 eV, hence again confirming the lower barriers for the larger K ions. While it is well known that K-GICs in general show lower diffusion barriers than Li-GICs^31,47,64–67^ the reason was typically ascribed to the differences in layer spacing caused by the ion sizes. Interestingly, at low AM concentrations, the overall finding that K ions diffuse much faster remains valid, whereas the layer spacing is only increased locally by the introduced distortion. However, this distortion seems large enough to still facilitate the jump to the neighboring empty sites.

### 3.4 Impact of Impurities on the Intercalation Process

As discussed above, the relative strength of the vdW interaction and AM-C bonds depends on the lateral size and the corresponding AM concentration. In the case of K, our findings clearly show that intercalation up to a certain K concentration is energetically unfavorable. Hence, it is anticipated that for the initial intercalation of K in graphite, additional driving forces will be beneficial. One way to provide driving forces for AM intercalation is the introduction of structural defects.^36,68^ The incorporation of heteroatoms, on the other hand, may also offer an additional way to tailor the carbon framework such that the early steps of intercalation are facilitated.^37,38^ Experimentally, doping strategies have been attracting certain interest and different doping elements such as for instance B, Si, or S have been proven to be able to increase the AM storage capabilities, while they are also observed to improve additional properties such as intercalation kinetics, structural stability or electronic conductivity.^69–74^ Heteroatom doping of graphite typically affects the electronic structure or the geometry of the system and can thereby result in favorable energetics for AM intercalation. Allowing facile incorporation into graphite, elements in the vicinity of graphite (B, Si, Sn, N, and S) were considered in the present work. In principle, boron can be expected to act as an electron acceptor, whereas Si and Sn, due to their size, are expected to introduce distortions into the system. Nitrogen, on the other hand, will act as an electron donor and, therefore, is not expected to be beneficial for AM intercalation. Finally, OH, which is often present in graphitic materials, and sulfur, an element frequently studied in combination with graphite, have been investigated. To show how heteroatom doping can affect the energetics of early-stage intercalation, we investigated the impact of the above-discussed impurities by incorporating them into the two-layer-based C_144_ bulk model system. For all impurities, except for the case of OH, a carbon atom was simply substituted with the doping element. The OH molecule, on the other hand, was placed on top of a carbon atom, thus forming a C–O–H moiety. It should be noted that the considered structures, apart from negligible distortions, remain AB stacked. To assess the impact of the doping elements, the intercalation energy, in analogy to eq[Disp-formula eq1] , was determined.

3 $$E_{\mathrm{int}}=E_{\mathrm{def}+\mathrm{AM}}-\left(E_{\mathrm{def}}+E_{\mathrm{AM}}\right)$$

Here, *E*_def+AM_ is the total energy of the graphitic (impurity containing) system after the insertion of one AM atom, and *E*_def_ is the energy of the AM-free layers, whereas *E*_AM_ is the energy of the AM in the bulk metal phase. In general, the obtained intercalation energies indicate that impurities (except nitrogen) can stabilize the intercalation at low AM concentrations (see Table 2 and Figure 6d). Here, it has to be pointed out that—similar to the case of undoped graphite (see Table S1)—small differences in intercalation energy might be observed at low AM content when model systems with an increased number of graphitic layers are considered. In particular, the intercalation of Na and K in the low concentration limit, which is unstable for pristine graphite, becomes favorable when impurities are considered.

**Table 2:** Intercalation Energy *E*_int_ (in eV) for Li, Na, and K Atoms in a Two-Layer-Based Bulk Model System (C_144_) Obtained for Different Impurities (B, Si, Sn, N, S, and OH), Mono-Vacancy Defect (MV), and also for Pristine Graphite (G)

Impurities	B	Si	Sn	N	S	OH	MV	G
Li	–1.24	–1.04	–1.91	–0.12	–0.31	–1.44	–1.45	–0.20
Na	–0.52	–0.69	–1.93	0.60	–0.09	–1.09	–0.54	0.59
K	–0.05	–0.27	–0.63	1.01	0.10	–0.66	–0.33	0.93

**Figure 6:**
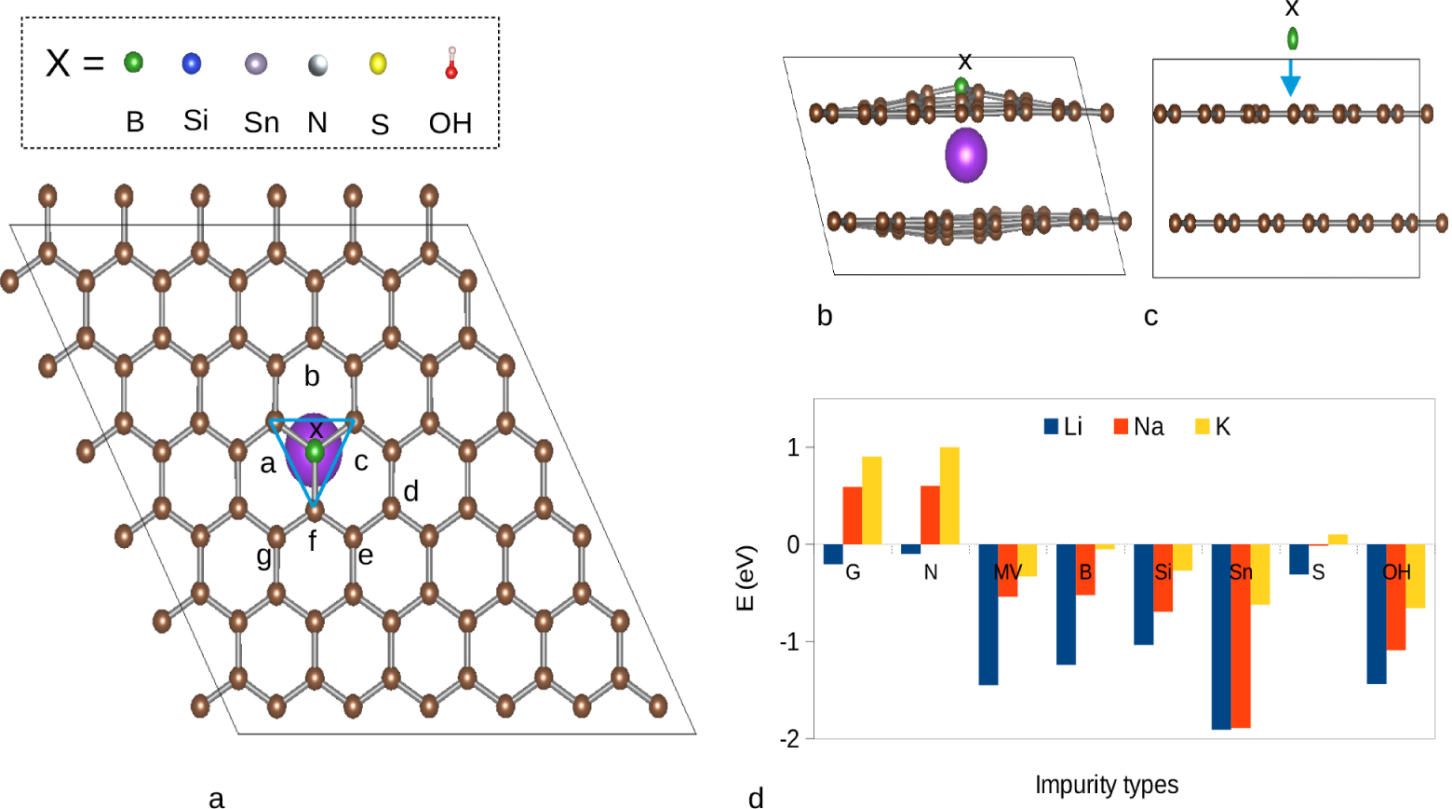
Different types of impurities in the graphitic system. (a) Top view of the model system (x = B, Si, Sn, N, S, and OH), where the AM atoms are shown on top of the impurity site, with the possible final sites marked by different letters, (b) side view of the model system after intercalation, (c) side view of the model system before intercalation and (d) intercalation energy *E*_int_ (in eV) for Li, Na, and K atoms in the two-layer based model (C_144_) near the impurity sites (see Table 2). G represents pristine graphite without defect and impurity.

Apart from altering the energetics, the impurities also have an impact on the equilibrium location of the AM atoms. In all cases, the intercalated AM atoms have originally been positioned at the center of a honeycomb structure that includes the impurity, denoted by the letter c (see Figure 6a). This corresponds to the AM positions determined for pristine graphite, i.e., with the AM located on top of the center of a six-membered ring of one graphene sheet and directly below a carbon atom of the other.

When B doping is considered, the geometry of the system is not significantly affected due to the comparable atomic sizes of boron and carbon. In fact, B–C bonds (1.48 Å) are only slightly increased compared to C–C bonds (1.43 Å) in pristine graphite. The AM intercalation process, on the other hand, is significantly stabilized by the presence of boron, such that Na (−0.52 eV) and K (−0.05 eV) intercalation also become energetically favorable. This stabilization is related to the interaction of boron and the intercalants. In the case of Na and K, the AM atoms prefer to sit on top of the B impurity, while for the Li atom, the c site (hexagon, beside the impurity) is more favorable. Whereas the intercalant-free system is essentially undistorted, the intercalation of AM atoms results in a curving of the graphitic planes, as in the case of pristine graphite. This is also visible in the increased B–C bond length, which amounts to 1.49, 1.51, and 1.51 Å for Li, Na, and K, respectively. By introducing Si as an impurity, the graphitic planes get slightly curved, which is a consequence of the increased atomic size of Si compared to C. In addition, a change in bond length is observed, with the Si–C bonds (1.72 Å) being longer than a pristine C–C bond. Despite the fact that Si and C have the same valence, the all intercalated AM atoms migrate on the top of the impurity while they are at the center of a hexagon in the adjacent layer. The Si–C bonds further increase from 1.72 Å in the intercalant-free system to 1.77–1.78 Å for AM intercalation. As for the pristine and B-doped systems, the curvature after intercalation scales with the AM size. From an energetic point of view, the AM intercalation is stabilized by the Si impurity, with K and Na again showing a negative intercalation energy. The stabilization effect is similar to the case of boron; however, it is a bit more pronounced for Na and K (see Figure 6d). Similarly, the introduction of isovalent Sn impurities creates a distorted graphitic plane, which is again due to the increased atomic size of Sn and the significantly larger Sn–C bond length (from 1.43 to 2.05 Å). Also for this case, all AM atoms migrate to the top of the impurity, while they are at the center of a hexagon in the adjacent layer. Again, as in the previous cases, the AM intercalation results in an intercalant size-dependent curvature of the system. The intercalation energies for Li, Na, and K amount to −1.91 eV, −1.93 eV, and −0.63 eV, thus showing the most pronounced stabilization of the intercalation process. For N doping, the N–C bonds are somewhat reduced (1.41 Å) as compared to the C–C bonds in pristine graphite (1.43 Å), but this tiny difference has no discernible impact on the system geometry. The Na and K intercalation remains unfavorable in contrast to the previously mentioned impurities (see Figure 6). This is because nitrogen acts as an electron donor, which reduces the effectiveness of electron transfer from intercalants to graphite. Furthermore, N doping does not substantially alter the system geometry, such that there is no additional driving force for intercalation. In terms of the AM locations, K atoms essentially stay inside the center of the hexagon in which they are initially positioned, while Li and Na shift to an off-centered position. When considering sulfur impurities, the S atom actually moves out of the graphitic plane, with the length of the S–C bonds amounting to 1.73 Å. During intercalation, the curvature increases according to the AM atom sizes. However, while the Li atom remains in its original position at the center of the honeycomb, Na and K atoms move to an off-center site (see Figure 6). The intercalation energies for Li, Na, and K amount to −0.31 eV, −0.09 eV, and 0.21 eV, respectively, meaning that K intercalation remains unfavorable. Under intercalation, the S–C bonds increase slightly to 1.74–1.76 Å.

Finally, with regard to the OH impurity, the intercalant-free system already becomes significantly curved, with the maximal layer spacing increasing from 3.39 to 4.22 Å. This results in a decreased energetic penalty for AM intercalation and, hence, a large energetic stabilization for Li (−1.44 eV), Na (−1.09 eV), and K (−0.66 eV). While the Li atom travels to the f site, Na and K migrate to the d and g sites, respectively. These sites correspond to positions on top of carbon atoms in the impurity plane and in the center of a hexagon in the second graphitic plane. It should be noted that adding OH impurities already induces curvature in the system, which is further amplified when AM atoms are intercalated (see Figure S4). This increase is accompanied by changes in the O–C bond length from 1.49 to 1.57 Å (1.60 and 1.60 Å) for Li (Na and K), whereas the C–C bonds are only slightly affected (∼1% variation).

Finally, to gain additional insight, we compare the impact of impurities and typically observed structural defects. Interestingly, the impact of a monovacancy (MV) was found to result in a stabilization of the intercalation process that is similar to the Si and B impurities, with the intercalation energies for Li, Na, and K amounting to −1.45 eV, −0.54 eV, and −0.33 eV, respectively.

Hence, stabilization of the early-stage intercalation, which is highly desirable for K (and Na), can be achieved by incorporating impurities or defects (or combinations thereof) into graphitic carbon. However, at the same time, excessively strong bonding of AM atoms may result in their permanent trapping, corresponding to irreversible capacity loss. Such a capacity loss may, to some extent, be tolerated, as it could be accompanied by a positive impact on the overall intercalation process, e.g., improving the intercalation kinetics and hindering AM plating. On the other hand, it would, of course, be desirable to design the best-performing anode material. Hence, the types of defects and impurities, as well as their ratios, should be controlled, as they are critical factors for optimizing carbon-based anode materials. While the incorporation of defects and impurities is possible—e.g., by creating soft or hard carbon materials from different organic precursors—the control of the resulting structures on the micro- and nanoscale remains a challenge that needs to be overcome.

## 4 Conclusion

In this work, we investigated the early stages of intercalation for Li, Na, and K in graphite-based model systems. Our findings reveal that the intercalation processes for Li and K are fundamentally different. Due to the small size of Li ions, the intercalation process does not result in significant distortions of the graphitic planes and, moreover, is energetically favorable from the beginning. For K intercalation, the large ion size results in significant distortions of the graphitic layers and a competition between vdW forces and ionic K–C bonds. This results in K intercalation compounds with a low K content being energetically unfavorable. In fact, only when a certain density of K atoms between graphitic layers is reached does the compound become energetically favorable. To achieve this, the extremely low diffusion barriers for K in AB-stacked graphite are highly beneficial. As a consequence, the initial steps for Li and K intercalation are different, resulting in a random distribution of Li atoms, while K atoms prefer to fill one layer in a given graphitic domain. The differences in the energetics of early-stage intercalation may hence also explain the higher risk of plating and the increased resistance observed for KIBs with graphitic anodes. Furthermore, the energetics of the first intercalation steps may also serve as an explanation for the sluggish kinetics observed despite the fact that experimental and computational studies show lower diffusion barriers for K compared to Li in graphite.^64^ We further confirmed the lower diffusion barriers for K-GICs compared to Li-GICs, which, due to the large distortion caused by K insertion, seems counterintuitive at first glance. However, these distortions are spatially extended such that the spacing of the graphite atoms on neighboring planes is still strongly increased, which in turn facilitates diffusion.^75^

Lastly, we investigated the impact of heteroatom doping on the energetics of the early-stage intercalation process. These, along with defects, can stabilize the early-stage K intercalation (and also Na-intercalation) and may thus help to overcome limitations such as K-plating and sluggish kinetics in graphitic anodes. These findings, together with the gained insights into the differences in the intercalation processes, may be applied to designing anode materials with improved performance.

## Supplementary Material



## Notes

Additional structure and NEB images and information on the three-layer based bulk system (PDF)
