# Supplementary material for: Uncovering the Early-Stage Intercalation Mechanism in Graphite-Based Anode Materials
Source: ACS Appl Mater Interfaces. 2025 May 28;17(23):33965–74. doi: 10.1021/acsami.5c04287 (PMC12163928; doi:10.1021/acsami.5c04287)
Supplement: Supplementary file 1 [file am5c04287_si_001.pdf]

# Supporting Information
# Uncovering the early stage intercalation mechanism in graphite-based anode materials.

Jafar Azizi,*,† Axel Groß,*,† and Holger Euchner*,‡

†*Institute of Theoretical Chemistry, Ulm University, D-89081 Ulm, Germany*

‡*Institute of Physical and Theoretical Chemistry, University of Tübingen, 72076 Tübingen, Germany*

E-mail: jafar-1.azizi-shoushbolaghi@uni-ulm.de; axel.gross@uni-ulm.de; holger.euchner@uni-tuebingen.de

Table S1: Intercalation energy $E_{int}$ (in eV) for Li, Na, and K atoms for the 3-layer based bulk system and according to the different supercell sizes.

| Models | $C_{24}$ | $C_{54}$ | $C_{96}$ | $C_{150}$ | $C_{216}$ |
|---|---|---|---|---|---|
| Li | -0.236 | -0.290 | -0.270 | -0.236 | -0.243 |
| Na | 0.063 | 0.053 | 0.359 | 0.467 | 0.572 |
| K | -0.340 | -0.199 | 0.435 | 0.958 | 1.217 |

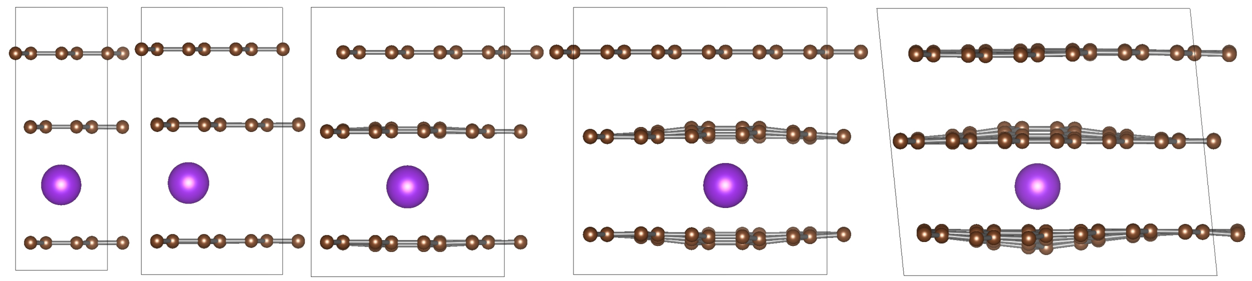

a b c d e

Figure S1: Schematic representation (side view) of the different supercell sizes used to model the AM-atom intercalation, showing the distortions introduced by K atoms in (a) $KC_{24}$, (b) $KC_{54}$, (c) $KC_{96}$, (d) $KC_{150}$ and (e) $KC_{216}$.

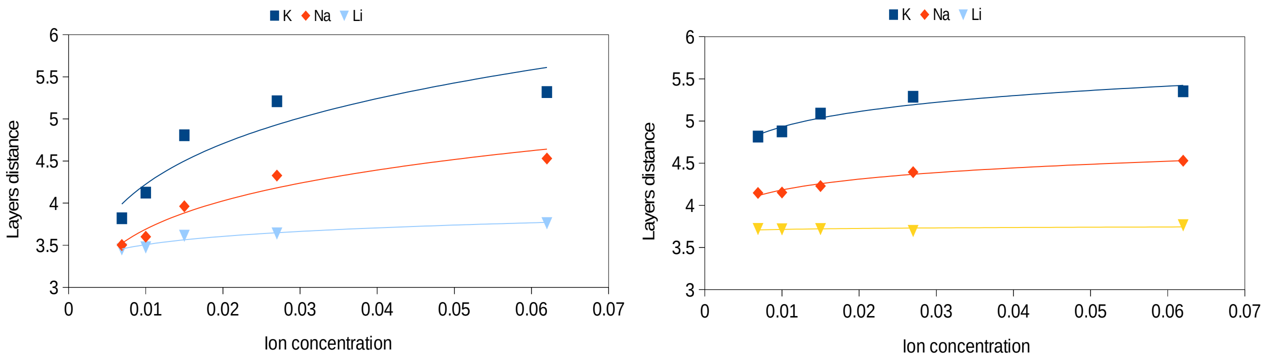


a b

Figure S2: Graphite layer distance for different supercell sizes of the 3-layer based bulk system ( $AMC_{N\times N\times 3}$, AM= Li, Na, and K, N= 2, 3, ..., 6). (a) Graphite layer distance far from the intercalant and (b) layer distance at the intercalant site.

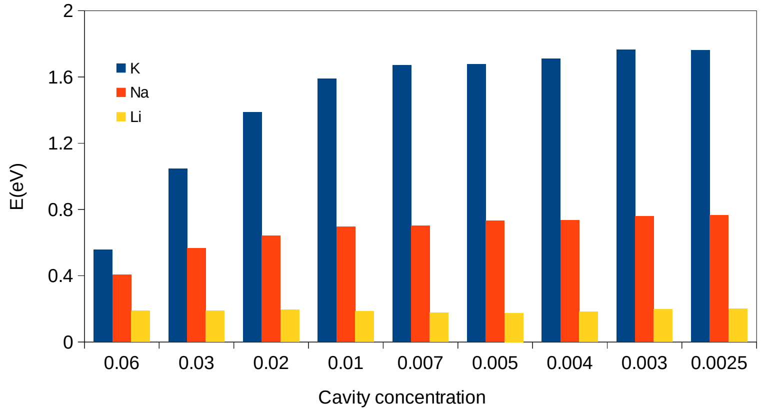


Figure S3: Intercalation energy $E_{int}$ (in eV) of single AM (Li, Na, and K) atoms for different lateral supercell sizes and hence different AM concentrations in a three layer based bulk system.

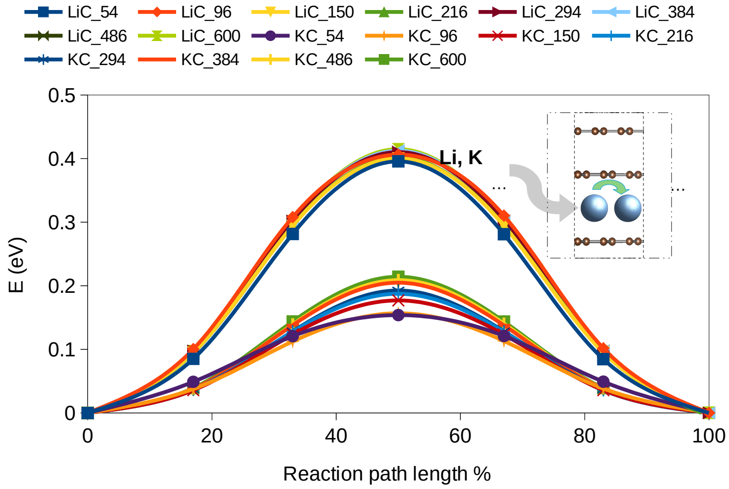


Figure S4: Minimum energy path for the three layer based bulk system with different lateral supercell size ($AMC_{N\times N\times 3}$, AM= Li, K, and N= 3, 4, 5, 6, 7, 8, 9, 10).

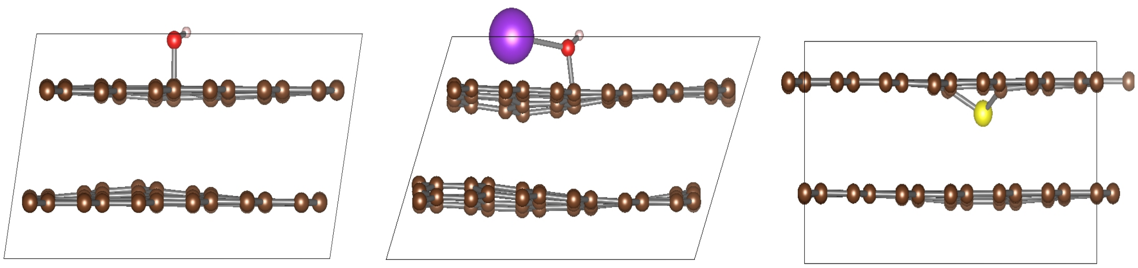

a b c

Figure S5: Adding an OH impurity introduces a certain curvature in the considered model system. The OH containing structures (a) before and (b) after the AM intercalation are depicted. In (c) the impact of adding a sulfur impurity is shown.
